# Pay Your Debts; or, the Lace Vail

**Published:** 1861-07

**Authors:** 


### PAY YOUR DEBTS; or, THE LACE VAIL.

A STORY went the round of the public papers, within two years, that a young woman was employed to prepare a lace vail for the approaching marriage of the daughter of royalty. Later on, she applied for her pay, but from some cause or other, she failed, after repeated efforts, to accomplish her object. She determined to tell her story to the ears of royalty itself, but her pressing necessities, the length of the road she had to walk, the rudeness and rebuffs she had to encounter, so weighed upon her health and spirits, that she died. The debt was then paid, but atonement for the wrong can never be made this side the judgment. "The Bible is the poor man's friend," used to be a favorite saying of that great and good man, Dr. James W. Alexander, and one of a multitude of proofs thereof, is the merciful injunction of the Jewish economy, "The wages of him that is hired shall not abide with thee all night until the morning," and among the closing curses of the Old Testament, was that which was threatened the perjured equally with him who "should oppress the hireling in his wages," the widow and the fatherless. The writer knew on one occasion, a single bank-bill to pay debts in a single forenoon, amounting in the aggregate, to three hundred and forty dollars, at a time of great pecuniary pressure. To refuse to pay a just debt on any pretense, when the money is on hand, without making it satisfactory in some way, to the person to whom the money is due, is to commit a great injustice, an inexcusable wrong. If the narration above made, is wholly true, no blame is hereby charged to the noblest living queen, but to those about her who refused to allow the knowledge of the matter to come to her ears. An illustration of the truth under consideration, is found in the following, which sounds so much like fact, that we can not say it is not so.

"Sir, if you please, boss would like you to pay this little bill to-day," said for the tenth time, a half-grown boy in a dirty jacket, to a lawyer in his office.

The attorney at length turned round and stared the boy full in the face, as if he had been some newly discovered specimen, gave a long whistle, thrust his inky fingers first into one pocket and then into the other of his black cloth vest, and then gave another long whistle, and completed his stare at the boy's face.

"Ho, ha, hum! that bill, eh?" said the legal young gentleman, extending the tips of his fingers toward the well-worn bit of paper, and daintily opening it, looked at the contents.

"Hum! for capping and for heel-tapping, six shillings—for foxing, ten and sixpence, and other sundries, eh? So your master wants me to settle this bill, eh?" repeated the man of briefs.

"Yes, sir; this is the nineteenth time I have come for it, and I intend to knock off at twenty, and call it half a day."

"You're an impudent boy."

"I's always impudent to lawyers, coz I can't help it—it's catchin'."

"You've got your eye-teeth cut, I see."

"That's what boss sent me for, instead of the 'prentices as was gettin' their teeth cut. I cut mine at nine months old with a hand-saw. Boss says if you don't pay the bill, he'll sue you."

"Sue *me?* I'm a lawyer!"

"It makes no odds. Lawyer or no lawyer, boss declares he'll do it—so fork over."

"Declares he'll sue me?"

"As true as there is another lawyer in Filadelphy."

"That would be bad!"

"Wouldn't it?"

"Silence, you vagabond! I suppose I must pay this," muttered the attorney to himself. "It's not my plan to pay these bills. What is a lawyer's profession good for, if he can't get clear of paying his own bills? He'll sue me! 'Tis just five dollars. It comes hard, and he don't want the money. What is five dollars to him? His boy could have earned it in the time he has been sending him to me for it.—So your master will sue me for it if I don't pay?"

"He says he will do it, and charge you a new pair of shoes for me."

"Harkee; I can't pay you to-day, and so if your boss will sue me, just ask him to employ me as his attorney."

"You!"

"Yes; I'll issue the writ, have it served, and then you see I shall put the cost into my own pocket, instead of seeing it go into another lawyer's. So you see if I have to pay the bill, I'll make costs—capital idea!"

The boy scratched his head awhile, as if striving to comprehend this capital idea, and shook it doubtingly. "I don't know about this; it looks tricky. I'll ask boss, though, if as how you won't pay it no how without being sued."

"I had rather be sued, if he will employ me, boy."

"But who is to pay them costs—the boss?"

The lawyer looked all at once very serious, and gave one of those long whistles peculiar to him.

"Well, I'm a sensible man, truly. My anxiety to get the costs of suit blinded me to the fact that they were to come out of my own pocket before they could be safely put in. Ah! well, my boy, I suppose I must pay. Here's a five dollar gold piece; is the bill receipted? it's so dirty and greasy I can't see."

"It was nice and clean when boss gin it to me, and the writin' shined like Knapp's blackin'—it is torn so a dunnin' so much."

"Well, here's your money," said the man of law, taking a solitary five dollar gold piece from his watch fob; "now tell your master, Mr. Last, if he has any other accounts he wants sued, I'll attend to them with the greatest pleasure."

"Thank'ee, sir," answered the boy, pocketing the five; "but you are the only dunnin' customer boss has, and now you've paid up, he haint none but cash folks. Good day to you."

"Now, there goes five dollars that will do that fellow no good. I am in want of it, but he is not. It is five thrown away. It wouldn't have left my pocket but that I was sure that his patience was worn out and costs would come of it. I like to get costs, but I can't think a lawyer has any thing to do with paying them."

As Peter Chancery did not believe, in his own mind, that paying his debt to Mr. Last was to be any benefit to him, and was of opinion that it was money thrown away, let us follow the fate of those five dollars through the day.

"He has paid," said the boy, placing the money in the master's hand.

"Well, I'm glad of it," answered Mr. Last, surveying the money through his glasses, "and it's a half-eagle, too. Now run and pay Mr. Furnace," as the boy delivered his errand and the money. "I was just wondering where I could get five dollars to pay a bill that is due to-day. Here, John," he called to one of his apprentices, "put on your hat, and take this money to Capt. O'Brine, and tell him I came within one of disappointing him, when some money came in I didn't expect."

Capt. O'Brine was on board his schooner at the next wharf, and with him was a seaman with a hat in his hand, looking very gloomy as he spoke with him.

"I'm sorry, my man, I can't pay you—but I have just raised and scraped the last dollar I can get above water, to pay my insurance money to-day, and have not a copper left in my pocket to jingle, but keys and old nails."

"But I am very much in need, sir; my wife is failing, and my family are in want of a good many things just now, and I got several articles at the store, expecting to get money of you to take them up as I went along home. We hain't in the house no flour, nor tea, nor——"

"Well, my lad, I'm sorry. You must come to-morrow. I can't help you unless I sell my coat off my back, or pawn my schooner's kedge. Nobody pays me."

The sailor who had come to get advance of wages, turned away sorrowfully, when the apprentice-boy came up and said in his hearing:

"Here, sir, is five dollars Mr. Furnace owes you. He says when he told you he couldn't pay your bill to-day, he didn't expect some money that came in after you left the shop."

"Ah! that's my fine boy. Here, Jack, take this five dollars, and come on Saturday and get the balance of your wages."

The seaman with a joyful bound took the piece, and touching his hat, sprung with a light heart on shore, and hastened to the store where he had already selected the comforts and necessaries his family stood so much in need of.

As he entered, a poor woman was trying to prevail upon the store-keeper to settle a demand for making his shirts.

"You had better take it out of the store, Mrs. Conway," he said to her, "really I have not half the amount of your bill to-day, and I don't expect to. I have to charge every thing, and no money comes in."

"I can't do without it," answered the woman earnestly, "my daughter is very ill and in want of every comfort; I am out of firewood, and indeed I want many things which I have depended on this money to get. I worked night and day to get your shirts done."

"I'm very sorry, Mrs. Conway," said the store-keeper, look- into his money-drawer; "I've not five shillings here—and your bill is five dollars and ninepence."

The poor woman thought of her invalid child and wrung her hands.

"A sailor was here awhile ago, and selected full five dollars worth of articles here on the counter and went away to get his wages to pay for them, but I question if he comes back. If he does and pays for them, you shall have your money, madam."

At this instant Jack made his appearance at the door.

"Well, shipmate," said he, in a tone much more elevated than when he was discovered speaking with the captain, "well, my hearty, hand over your freight. I've got the documents, so give us possession;" and displaying his five dollar piece he laid hold of the purchases. The store-keeper examining and seeing that the money was good, bade him take them with him; and then, sighing as he took another and a last look at the piece, he handed it to the poor widow, who with a joyful smile received it from him and hastened from the store. In a low and very humble tenement, near the water, was a family of poor children, whose appearance exhibited the utmost destitution. On a cot-bed lay a poor woman, ill and emaciated. The door opened and a man in coarse, patched garments, entered with a wood-saw and a horse, and laid them down by the door-side and approached the bed.

"Are you any better, dear?" he asked in a rough voice, but in the kindest tones.

"No; have you found work? If you could get me a little nourishing food. I could regain my strength."

The man gazed upon her pale face a moment, and again taking up his horse went out. He had not gone far before a woman met him, and said she wished him to follow and saw some wood for her. His heart bounded with hope and gratitude, and he went after her to her dwelling, an abode little better than his own for poverty, yet wearing an air of comfort. He sawed the wood, split and piled it, and received six shillings, with which he hastened to a store for necessaries for his sick wife, and then hurried home to gladden her heart with the delicacies he had provided. Till now he had had no work for four days, and his family had been starving, and from this day his wife got better, and was at length restored to his family and to health, from a state of weakness which another day's continuation would probably have made fatal.

These six shillings, which did so much good, were paid him by the poor woman from the five dollars she had received from the store-keeper, and which the sailor had paid him. The poor woman's daughter was also revived and ultimately restored to health, and was lately married to a young man who had been kept three years absent, and returned true to his troth. But for the five dollars which had been so instrumental in her recovery, he might have returned to be told that she whose memory had been so long the polar star of his heart had perished.

So much good did the five dollar piece do, which Peter Chancery, Esq., so reluctantly paid to Mr. Last's apprentice-boy, though little credit is due to this gentleman for the result that followed. It is thus Providence often makes bad men the instrument of good to others. Let this little story lead those who think a "small bill" can stand because it is a small bill, remember how much good a five dollar piece has done in one single day, and that in paying one bill they may be paying a series of twenty bills, and dispensing good to hundreds around them.

